# Discrimination between clinical significant and insignificant prostate cancer with apparent diffusion coefficient – a systematic review and meta analysis

**DOI:** 10.1186/s12885-020-06942-x

**Published:** 2020-05-27

**Authors:** Hans-Jonas Meyer, Andreas Wienke, Alexey Surov

**Affiliations:** 1 Department of Diagnostic and Interventional Radiology, University of Leipzig, Leipzig, Germany; 2 Institute of Medical Epidemiology, Biostatistics, and Informatics, Martin-Luther-University Halle-Wittenberg, Halle (Saale), Germany

**Keywords:** Meta analysis, Systematic review, Prostate cancer, DWI, Gleason score

## Abstract

**Background:**

Prostate MRI has become a corner stone in diagnosis of prostate cancer (PC). Diffusion weighted imaging and the apparent diffusion coefficient (ADC) can be used to reflect tumor microstructure. The present analysis sought to compare ADC values of clinically insignificant with clinical significant PC based upon a large patient sample.

**Methods:**

MEDLINE library and SCOPUS databases were screened for the associations between ADC and Gleason score (GS) in PC up to May 2019. The primary endpoint of the systematic review was the ADC value of PC groups according to Gleason score. In total 26 studies were suitable for the analysis and included into the present study. The included studies comprised a total of 1633 lesions.

**Results:**

Clinically significant PCs (GS ≥ 7) were diagnosed in 1078 cases (66.0%) and insignificant PCs (GS 5 and 6) in 555 cases (34.0%). The pooled mean ADC value derived from monoexponenantially fitted ADC_mean_ of the clinically significant PC was 0.86 × 10^− 3^ mm^2^/s [95% CI 0.83–0.90] and the pooled mean value of insignificant PC was 1.1 × 10^− 3^ mm^2^/s [95% CI 1.03–1.18]. Clinical significant PC showed lower ADC values compared to non-significant PC. The pooled ADC values of clinically insignificant PCs were no lower than 0.75 × 10^− 3^ mm^2^/s.

**Conclusions:**

We evaluated the published literature comparing clinical insignificant with clinically prostate cancer in regard of the Apparent diffusion coefficient values derived from magnetic resonance imaging. We identified that the clinically insignificant prostate cancer have lower ADC values than clinically significant, which may aid in tumor noninvasive tumor characterization in clinical routine.

## Background

Multiparametric magnetic resonance imaging (mpMRI) has become a corner stone of diagnosis in prostate cancer (PC) in a cost effective and highly accurate manner [1–4]. A great concern in PC treatment is possible over-diagnosing and over-treatment due to very different biological behaviors of PC, discriminated using Gleason score (GS) [5–7]. GS is still one of the most important prognostic features in prostate cancer [6]. So, a cancer with a GS 6 or lower is considered as a clinically insignificant cancer, which will most likely not result in cancer related death. Therefore, it can be treated in some cases with clinical surveillance [5]. However, PC with a GS of 7 and higher is clinically significant and is associated with tumor related morbidity/mortality [8].

In clinical routine mpMRI is very beneficial due to the high negative predictive value [1]. However, mpMRI can also detect more lesions than conventional diagnostic work flow, which might result in more insignificant cancers [9].

Diffusion-weighted imaging (DWI) is an important sequence of mpMRI. DWI reflects free water movement in tissues [10]. Furthermore, restriction of free water movement in tissues can be quantified by apparent diffusion coefficient (ADC) [10]. ADC is associated with histological features, which restrict diffusion of water molecules, like cell count and protein concentration in the extracellular space [11, 12]. Thus, ADC may aid in discrimination of several tumors. Previously, numerous studies reported that malignant tumors have significantly lower ADC values compared to benign lesions [13, 14]. PC had also lower ADC values in comparison to benign prostatic tissue [15]. Therefore, DWI is an established technique for detection of PC, especially in the peripheral zone [3].

Besides diagnostic potential, DWI/ADC can also aid characterize prostatic tumors. So far, a recent meta-analysis showed that ADC values correlated inversely with GS [16]. In detail, a correlation coefficient of r = − 0.45 between ADC and GS was reported in all PCs [16]. Furthermore, it was stronger in PC located in the peripheral zone (r = − 0.48) in comparison to PCs arose in the transitional zone (r = − 0.22). Presumably, ADC may discriminate low risk PCs from high risk tumors. However, published data above are inconsistent and based on small single center studies.

The purpose of the present systematic review and meta-analysis was to compare ADC values between clinically significant and non-significant PCs according to GS in a large patient sample.

## Methods

### Data acquisition

**Fig. 1:**
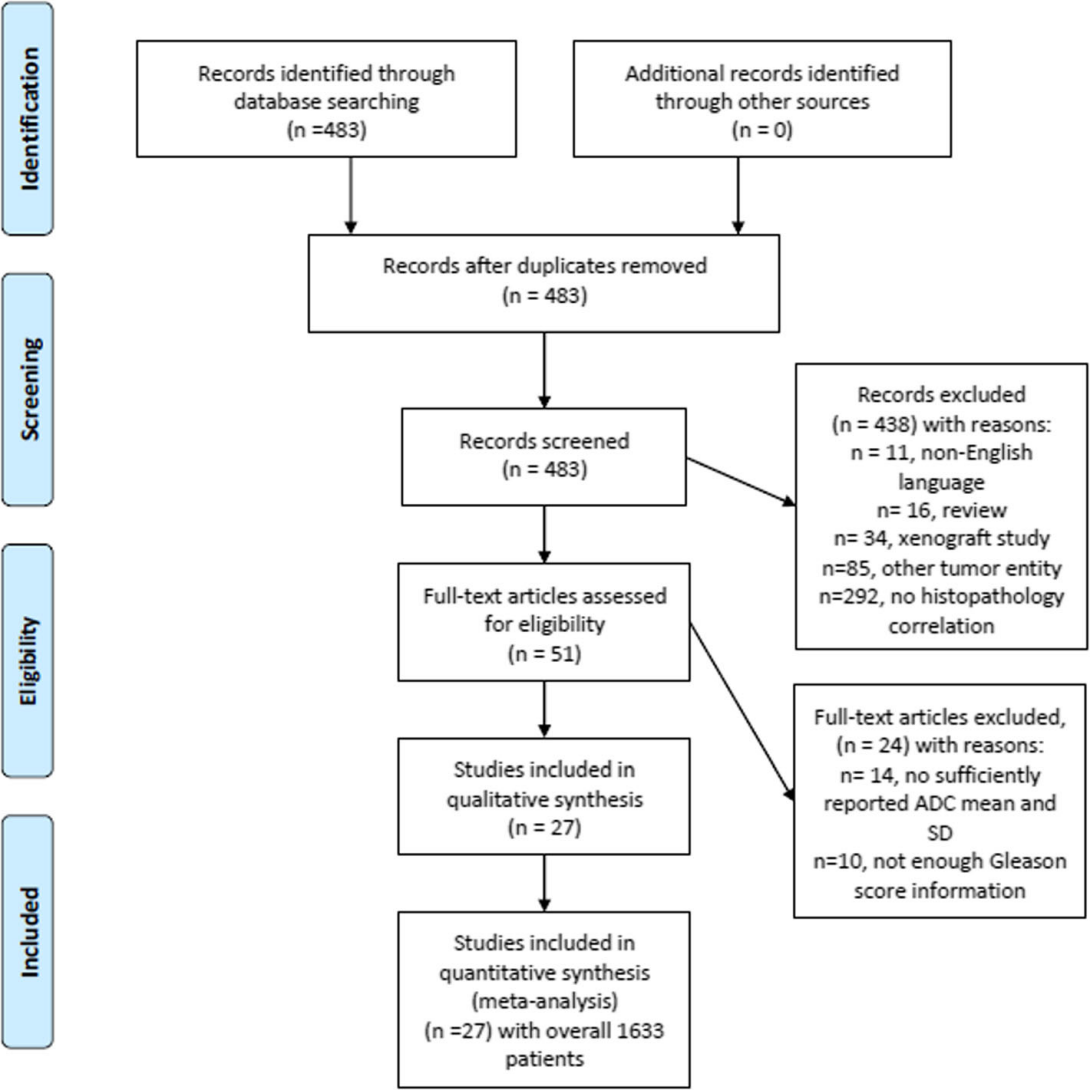
PRISMA flow chart. An overview of the paper acquisition. Overall, 27 articles comprising 1633 patients were suitable for the analysis

MEDLINE library, EMBASE and SCOPUS databases were screened for the associations between ADC and Gleason score in PC up to May 2019. The paper acquisition is summarized in Fig. 1.

The following search words were used: “prostate cancer OR prostatic carcinoma OR prostatic cancer OR prostate carcinoma AND DWI OR diffusion weighted imaging OR ADC OR apparent diffusion coefficient AND Gleason score AND Gleason”.

The primary endpoint of the systematic review was the ADC value of PC groups according to Gleason score.

Studies (or subsets of studies) were included, if they satisfied all the following criteria: (1) patients with PC confirmed by histopathology, (2) mpMRI with DWI sequence quantified by ADC values, and (3) reported ADC value according to GS.

Exclusion criteria were (1) systematic review, (2) case reports, (3) treatment prediction or histopathology performed after treatment, (4) non-English language, and (5) experimental (xenograft or animals model) studies.

The Preferred Reporting Items for Systematic Reviews and Meta-Analyses (PRISMA) statement was used for the analysis [17]. In total 26 studies were suitable for the analysis and included into the present study [18–43].

### Quality-assessment

**Fig. 2:**
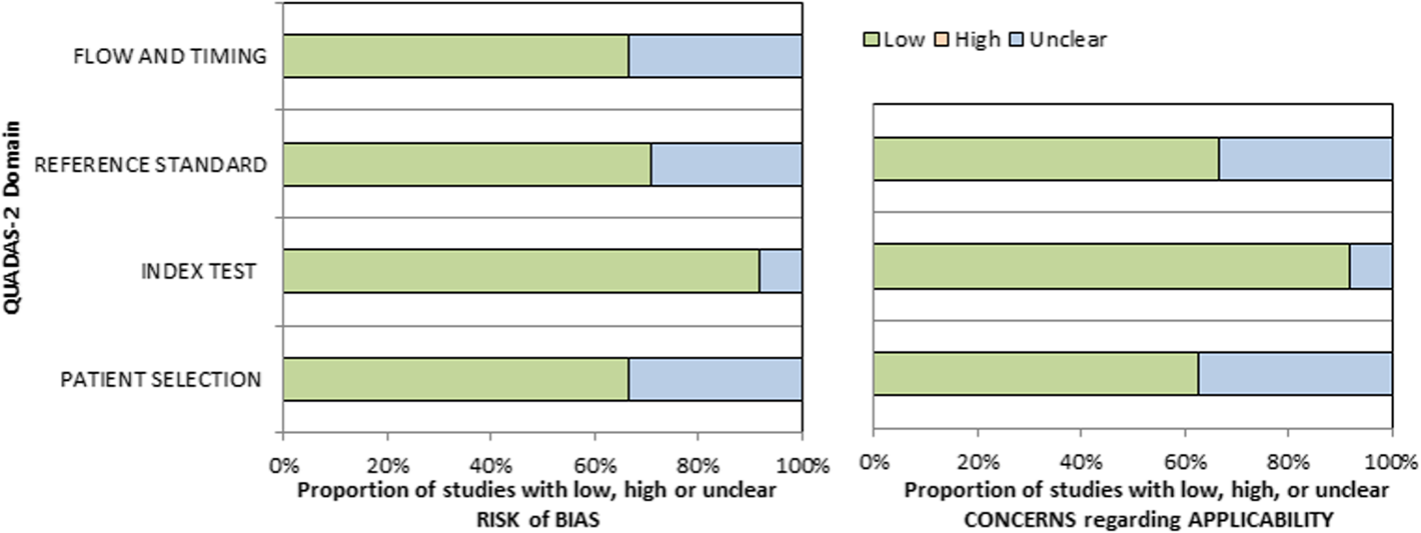
QUADAS-2 quality assessment of the included studies. Most studies showed an overall low risk of bias

The methodological quality of the acquired studies was independently evaluated by two readers (A.S. and H.J.M.) using the Quality Assessment of Diagnostic Accuracy Studies (QUADAS-2) instrument [44]. Results of QUADAS-2 assessments are shown in Fig. 2.

### Statistical analysis

The meta analysis was performed using RevMan 5.3 (2014; Cochrane Collaboration, Copenhagen, Denmark). Heterogeneity was calculated by means of the inconsistency index *I*^2^ [45, 46]. Finally, DerSimonian and Laird [47] random-effect models with inverse-variance weights were performed without any further correction.

## Results

Of the included 26 studies, 8 (30.7%) were of prospective and 18 of (69.3%) retrospective design. Different 1.5 T scanners were used in 7 (26.9%) studies and 3 T scanners in 19 (73.1%) studies. In 7 studies (26.9%) an additional endorectal coil was used. In 8 studies (30.7%) a bowel preparation was performed.

Regarding the QUADAS-2 assessments, most studies had a low risk of bias. For patient selection in 8 studies (29.6%) had an unclear risk of bias mainly based on not sufficiently reported inclusion and exclusion criteria of the patient sample. For the reference standard 7 studies (26.9%) had an unclear risk of bias due to insufficient reported histopathology standard or blinded reading of the pathologic specimen. Only small concerns were identified for the reported index tests (7.4% of studies with unclear risk of bias). For 8 studies 8 (29.6%) there was unclear risk of bias for flow and timing due to not sufficiently reported time duration between biopsy and imaging of the patients. The same reasons were identified in the groups for applicability of the results.

In all studies, the diagnosis was confirmed by histopathology. The histopathological diagnosis and scoring of PC was made on specimen after radical prostatectomy in 14 studies (53.8%), in 10 studies (38.5%) after transrectal ultrasound guided biopsy, and in 2 studies (7.7%) with both techniques.

The acquired 26 studies comprised a total of 1633 lesions. Clinically significant PCs (Gleason score 7 and higher) were diagnosed in 1078 cases (66.0%) and insignificant PCs (Gleason score 5 and 6) in 555 cases (34.0%).

**Fig. 3:**
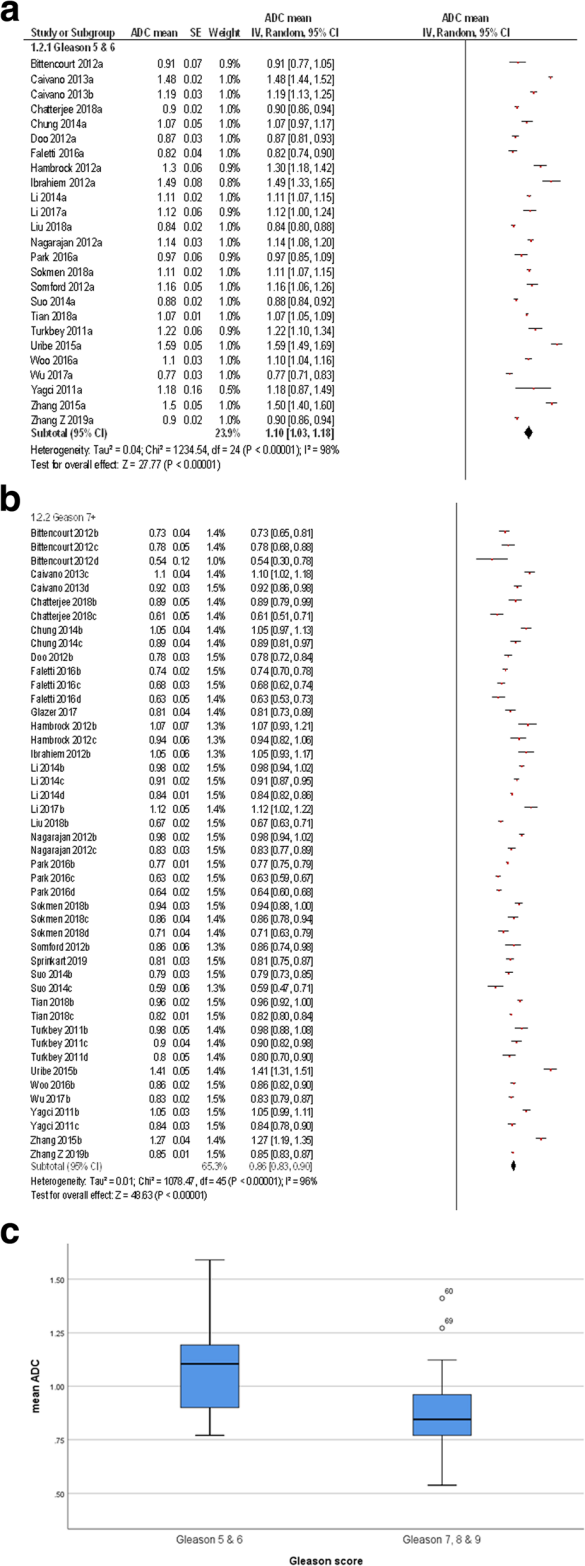
**a** Forrest plots of the mean apparent diffusion coefficients of clinical insignificant PC comprising Gleason score 5 and 6. The pooled mean ADC value was 1.10 × 10^− 3^ mm^2^/s [95% CI 1.03–1.18]. **b.** Forrest plots of the mean apparent diffusion coefficients of clinically significant PC comprising Gleason score 7 and higher. The pooled mean ADC value was 0.96 × 10^− 3^ mm^2^/s [95% CI 0.83–0.90]. **c.** Box plots of the mean ADC values of clinical insignificant and clinically significant PC. Clinical insignificant PC have lower ADC values than clinically significant PC.

The pooled mean ADC value of the clinically significant PC was 0.86 × 10^− 3^ mm^2^/s [95% CI 0.83–0.90, Tau^2^ = 0.01, Chi^2^ = 1078.47, df = 45, I^2^ = 96%] and the pooled mean ADC value of insignificant PC was 1.1 × 10^− 3^ mm^2^/s [95% CI 1.03–1.18, Tau^2^ = 0.04, Chi^2^ = 1234.54, df = 24, I^2^ = 98%]. Figure 3 shows the distribution of ADC values divided in clinically significant and insignificant PC.

**Fig. 4:**
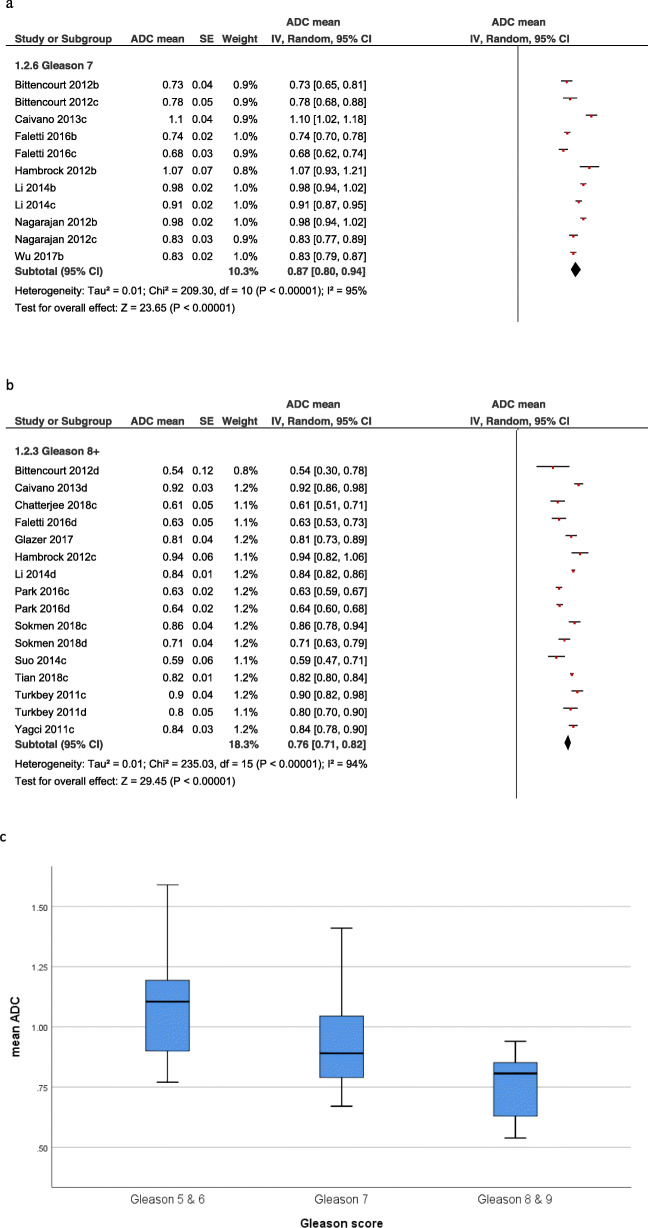
**a.** Forrest plots of the mean apparent diffusion coefficients of PC with Gleason score 7. The pooled mean ADC value was 0.87 × 10^− 3^ mm^2^/s [95% CI 0.80–0.94]. **b.** Forrest plots of the mean apparent diffusion coefficients of PC with Gleason score 8 and higher. The pooled mean ADC value was 0.76 × 10^− 3^ mm^2^/s [95% CI 0.71–0.82]. **c.** Box plots of the mean ADC values of clinical insignificant comprising Gleason score 5 and 6, Gleason score 7 and Gleason score 8 and 9 PC groups. There is a clear trend for higher Gleason score PC to have lower ADC values.

Thereafter, PCs were divided into subgroups according to the GS as follows: GS 5 and 6 (*n* = 555, 34.0%), GS 7 (*n* = 258, 15.8%), GS 8 (*n* = 42, 2.6%) and GS 9 (*n* = 30, 1.8%). The pooled mean ADC values of the subgroups were as follows: GS 5 + 6 = 1.1 × 10^− 3^ mm^2^/s [95% CI 1.03–1.18, Tau^2^ = 0.04, Chi^2^ = 1234.54, df = 24, I^2^ = 98%], GS 7 = 0.87 × 10^− 3^ mm^2^/s [95% CI 0.80–0.94, Tau^2^ = 0.01, Chi^2^ = 209.3, df = 10, I^2^ = 95%], and GS 8 and 9 = 0.76 × 10^− 3^ mm^2^/s [95% CI 0.71–0.82, Tau^2^ = 0.01, Chi^2^ = 235.03, df = 15, I^2^ = 94%] (Fig. 4).

**Fig. 5:**
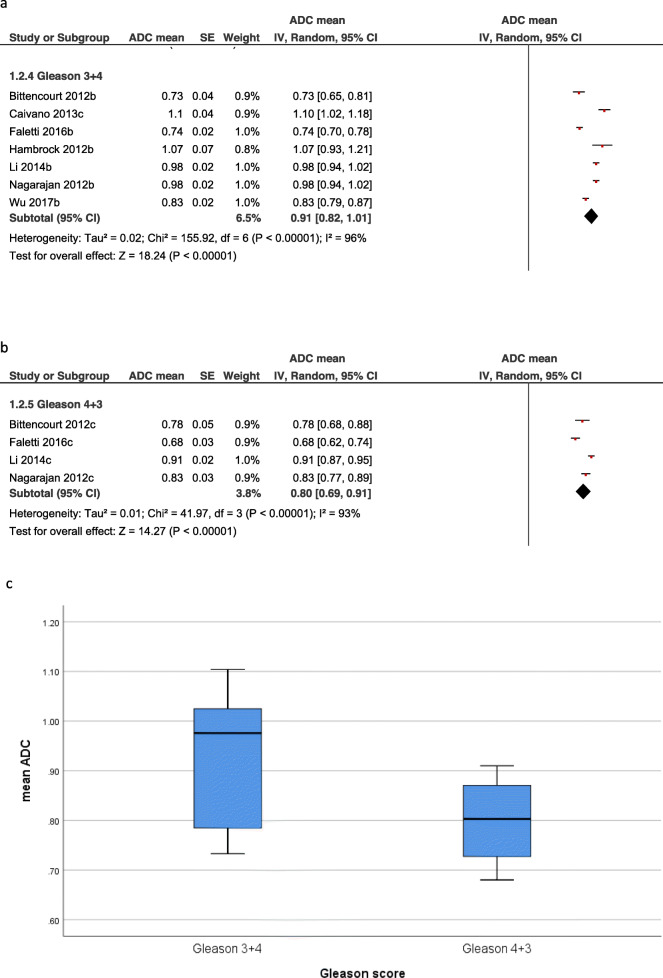
**a.** Forrest plots of the mean apparent diffusion coefficients of PC with Gleason score 3 + 4. The pooled mean ADC value was 0.91 × 10^− 3^ mm^2^/s [95% CI 0.82–1.01]. **b.** Forrest plots of the mean apparent diffusion coefficients of PC with Gleason score 4 + 3. The pooled mean ADC value was 0.80 × 10^− 3^ mm^2^/s [95% CI 0.69–0.91]. **c.** Box plots of the mean ADC values of Gleason score 3 + 4 and Gleason score 4 + 3. Gleason 4 + 3 PC have lower ADC values than Gleason score 3 + 4

Furthermore, the GS 7 group was divided into cancers with a primary GS 3 pattern with a sum of 3 + 4 and those with a primary GS 4 pattern with a sum of 4 + 3. GS 3 + 4 were total 7 studies with 170 lesions. The pooled mean ADC value was 0.91 × 10^− 3^ mm^2^/s [95% CI 0.82–1.01, Tau^2^ = 0.02, Chi^2^ = 155.92, df = 6, I^2^ = 96%]. GS 4 + 3 were total 4 studies with 88 lesions. The pooled mean ADC value was 0.80 × 10^− 3^ mm^2^/s [95% CI 0.69–0.91, Tau^2^ = 0.01, Chi^2^ = 41.97, df = 3, I^2^ = 93%] (Fig. 5).

### Subgroup analyses

To evaluate the high heterogeneity of the results, we performed subgroup analyses.

### Clinically insignificant PC

**Fig. 6:**
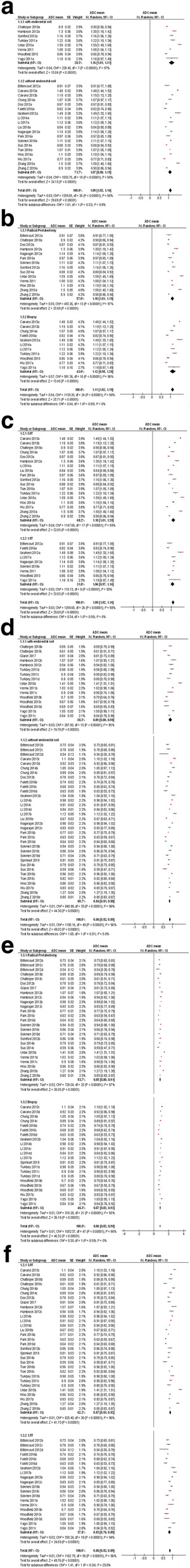
**a** Forrest plots of the mean apparent diffusion coefficients of the subgroup analysis of clinical insignificant PC in accordance to endorectal coil. The pooled mean ADC value was 1.16 × 10^− 3^ mm^2^/s [95% CI 1.01–1.31] for lesions investigated with endorectal coils and 1.07 × 10^− 3^ mm^2^/s [95% CI 0.98–1.15] for tumors without. **b**. Forrest plots of the mean apparent diffusion coefficients of the subgroup analysis of clinical insignificant PC in accordance to histopathology specimen. For PC investigated histopathologically after radical prostatectomy the pooled mean ADC value was 1.10 × 10^− 3^ mm^2^/s [95% CI 1.01–1.19] and it was 1.12 × 10^− 3^ mm^2^/s [95% CI 0.95–1.28] for lesions, which were classified based on bioptic specimens. **c**. Forrest plots of the mean apparent diffusion coefficients of the subgroup analysis of clinical insignificant PC in accordance to tesla strength. The pooled mean ADC value was 1.10 × 10^− 3^ mm^2^/s [95% CI 1.01–1.20] for lesions investigated by 3 T scanners and 1.06 × 10^− 3^ mm^2^/s [95% CI 0.97–1.16 for PC investigated by 1.5 T scanners. **d**. Forrest plots of the mean apparent diffusion coefficients of the subgroup analysis of clinically significant PC in accordance to endorectal coil. The pooled mean ADC value was 0.89 × 10^− 3^ mm^2^/s [95% CI 0.80–0.98] for lesions investigated with endorectal coils and 0.84 × 10^− 3^ mm^2^/s [95% CI 0.81–0.88] for PC investigated without endorectal coils. **e**. Forrest plots of the mean apparent diffusion coefficients of the subgroup analysis of clinically significant PC in accordance to histopathological specimens. For PC investigated histopathologically after radical prostatectomy the pooled mean ADC value was 0.85 × 10^− 3^ mm^2^/s [95% CI 0.80–0.91, Tau^2^ = 0.02, Chi^2^ = 728.04, df = 25, I^2^ = 97%], and it was 0.87 × 10^− 3^ mm^2^/s [95% CI 0.82–0.92, Tau^2^ = 0.01, Chi^2^ = 310.2, df = 21, I^2^ = 93%] for lesions investigated on bioptic specimens. **f**. Forrest plots of the mean apparent diffusion coefficients of the subgroup analysis of clinically significant PC in accordance to tesla strength. The pooled mean ADC value was 0.87 × 10^− 3^ mm^2^/s [95% CI 0.83–0.92] for PC investigated by 3 T scanners and 0.83 × 10^− 3^ mm^2^/s [95% CI 0.76–0.89] for tumors analyzed by 1.5 T scanners

The pooled mean ADC value of the clinically insignificant PC (GS ≤ 6) was 1.16 × 10^− 3^ mm^2^/s [95% CI 1.01–1.31, Tau^2^ = 0.04, Chi^2^ = 228.4, df = 7, I^2^ = 97%] in the studies that used endorectal coils and 1.07 × 10^− 3^ mm^2^/s [95% CI 0.98–1.15, Tau^2^ = 0.04, Chi^2^ = 1030.75, df = 18, I^2^ = 98%] in the reports without (Fig. 6a).

In PC that were investigated by histopathology after radical prostatectomy the pooled mean ADC value was 1.10 × 10^− 3^ mm^2^/s [95% CI 1.01–1.19, Tau^2^ = 0.03, Chi^2^ = 453.26, df = 13, I^2^ = 97%]. It was 1.12 × 10^− 3^ mm^2^/s [95% CI 0.95–1.28, Tau^2^ = 0.07, Chi^2^ = 581.36, df = 10, I^2^ = 98%] in tumors that were investigated after prostate biopsy (Fig. 6b).

Furthermore, the pooled mean ADC value was 1.10 × 10^− 3^ mm^2^/s [95% CI 1.01–1.20, Tau^2^ = 0.04, Chi^2^ = 1147.69, df = 17, I^2^ = 99%] for lesions investigated by 3 T scanners and 1.06 × 10^− 3^ mm^2^/s [95% CI 0.97–1.16, Tau^2^ = 0.02, Chi^2^ = 110.13, df = 8, I^2^ = 93%] for tumors investigated by 1.5 T scanners (Fig. 6c).

### Clinically significant PC

Regarding clinically significant PC the pooled mean ADC value was 0.89 × 10^− 3^ mm^2^/s [95% CI 0.80–0.98, Tau^2^ = 0.03, Chi^2^ = 287, df = 15, I^2^ = 95%] for investigations with endorectal coils and 0.84 × 10^− 3^ mm^2^/s [95% CI 0.81–0.88, Tau^2^ = 0.01, Chi^2^ = 840.88, df = 33, I^2^ = 96%] for studies without use of endorectal coils (Fig. 6d).

The pooled mean ADC value in PC analyzed histopathologically after radical prostatectomy was 0.85 × 10^− 3^ mm^2^/s [95% CI 0.80–0.91, Tau^2^ = 0.02, Chi^2^ = 728.04, df = 25, I^2^ = 97%]. It was 0.87 × 10^− 3^ mm^2^/s [95% CI 0.82–0.92, Tau^2^ = 0.01, Chi^2^ = 310.2, df = 21, I^2^ = 93%] for cases investigated histopathologically after prostate biopsy (Fig. 6e).

Regarding Tesla strength, the pooled mean ADC value was 0.87 × 10^− 3^ mm^2^/s [95% CI 0.83–0.92, Tau^2^ = 0.01, Chi^2^ = 825.4, df = 30, I^2^ = 96%] for PC investigated by 3 T scanners and 0.83 × 10^− 3^ mm^2^/s [95% CI 0.76–0.89, Tau^2^ = 0.02, Chi^2^ = 330.24, df = 19, I^2^ = 94%] for tumors investigated by 1.5 T scanners (Fig. 6f).

## Discussion

The present work is the first systematic review and meta-analysis comparing ADC values of clinically significant and insignificant PCs classified according to GS. Because it is based on a large cohort, it provides evident data regarding the quantitative analysis of DWI in distinguishing of different PCs.

GS is still one of the most important prognostic factors in PC to stratify patients employing a robust and durable method [6, 48]. So, GS is significantly associated with biochemical free survival [49]. As already mentioned, there is need to discriminate clinically insignificant PCs (GS 6 and lower), which are in almost every cases sufficiently treated with radical prostatectomy, whereas GS 7 and higher cancers are defined as clinically significant with a possibility of recurrence and tumor related death [48]. To predict GS non-invasively by mpMRI might be crucial because it is increasingly used in clinical routine. Thus, more cancers will be detected, which might result in over-diagnosing and over-treatment, when more clinically insignificant tumors are detected.

As reported previously, DWI/ADC can reflect tissue microstructure in several tumor entities, including PC [11]. In most studies, ADC inversely correlated with cellularity [11]. This is explained by the fact that the extracellular protons are mainly producing the MRI signal. Thus, in cell rich tumors, the extracellular water movement is lowered and correspondingly, the ADC value is also lowered.

However, it is also important to consider that DWI is sensitive on multiple spatial scales [50]. So, the time interval of the DWI measurement has an impact on how each water molecule is likely to encounter the tissue microstructure. For long diffusion times, structure heterogeneity on the smallest scales will be averaged and the signal attenuation will primarily be depended on large scale tissue structure features [50]. Moreover, the presented results depend on the used b-values in each study. Another important aspect is that the robustness of ADC values in clinical routine depends on fitting quality, repeatability of fitted parameters, robustness against measurement noise and clinically useful information [51]. Moreover, the present analysis only evaluated the monoexponential model to fit the ADC values. There are other methods, comprising non monoexponential models such as diffusion kurtosis imaging, which might better reflect microstructure of PC and better correlate with GS. However, there are some indications that the monoexponential model predicted the GS better with a higher repeatability compared to the intravoxel incoherent motion imaging model [52].

These facts might also be responsible for the large heterogeneity identified for the ADC values of the present study. We performed subgroup analyses but there were no substantial differences of ADC values obtained under different conditions like use of endorectal coiland tesla strength. Also no differences of ADC values were found between the tumors diagnosed after prostatectomy and PC diagnosed by prostate biopsy.

As reported previously, in PC, not only cell density is important, but also the glandular structure and formation of the tissue, which is also the most important factor for GS grading [6, 48, 49]. According to the literature, besides cellularity, ADC can also reflect other histopathological features in PC including proliferation index, vascular endothelial expression and hypoxia 1-alpha expression [53, 54]. In fact, it was unambiguously shown that ADC values are positively correlated with amount of glandular lumen with r = 0.688 and ADC values are negatively correlated to sole cell count (r = − 0.598) [53].

Consequently, ongoing research, showed weak to moderate inverse correlations between ADC values and GS, which further strengthened that ADC values are able to reflect tumor microstructure in a non-invasive way with possible translational benefit in daily clinical routine [16].

In a meta-analysis pooling 13 studies with 1107 tumor foci dated up to 2015, a sensitivity of 76.9% and a specificity of 77% was calculated for discrimination of clinically significant against insignificant based upon ADC values. In a subgroup analysis a higher sensitivity was identified for studies employing high b-values of 2000 s/mm^2^ [55].

The present meta-analysis showed that ADC values of different PCs distinct overlapped. However, clinically significant PC defined as PC with GS 7 and higher had lower ADC values than insignificant PCs. Moreover, the pooled ADC values of clinically insignificant PCs were no lower than 0.75 × 10^− 3^ mm^2^/s.

However, the present results cannot aid in proposing an ADC threshold for clinical routine due to differences in MRI technique in various instances [56, 57], mainly b-values, tesla strength and echo time. So, for every institution the ADC threshold needs to be evaluated.

There is recent literature suggesting that GS7 tumors include biological heterogeneous PCs. So far, GS 7 cancers can be estimated as 3 + 4 and 4 + 3 [58–61]. For the first group, the well differentiated cancer pattern is predominant. In contrast, for 4 + 3 lesions, the less differentiated pattern is predominant. This also might reflect different tumor behavior. For example, 4 + 3 cancers are more likely to be tumors with greater pathologic stage, and total tumor volume [58]. Our data corroborate the notion that GS7 cancers are heterogeneous in terms of their ADC values. In fact, GS 3 + 4 tumors had higher ADC values in comparison to GS 4 + 3 cancers. Presumably, ADC values are able to aid stratify GS 7 cancer, albeit further studies are needed to confirm these results.

Of note, in clinical routine the definition of clinically significant cancer is not only performed on GS alone but also the length and number of the positive biopsy core and the tumor volume of prostatectomy specimen. Moreover, seminal vesicle invasion and lymph node metastasis are key findings to define clinically significant cancer [62]. In the present analysis however only the GS could be evaluated to define clinically significant cancers.

Interestingly, some previous studies indicated that conventional imaging analysis by PIRADS scoring, a clinical used scoring system to predict the malignancy possibility, is not capable to discriminate between clinical significant and non-significant PC [63]. In PIRADS scoring, only a qualitative assessment based upon DWI, T2-weighted imaging, and contrast enhanced dynamic MRI [3]. ADC values are not quantitatively assessed in this system. Presumably, ADC values might harbor crucial information regarding GS in PC, which is not currently considered in clinical practice. In fact, Pierre et al. suggested that ADC quantification might aid in diagnosing of PC beyond the qualitative DWI assessment [64].

The present meta-analysis has several limitations to address. Firstly, it is mainly comprised of retrospective studies with possible known bias. Secondly, it was not possible to further stratify the patient samples according to tumor localization. Recently, a meta-analysis showed that cancers arising from transitional zone weaker correlated with GS, which might have an influence on the present analysis. Thirdly, we could not divide the patient sample according to biopsy and radical prostatectomy grading. It was shown that both methods might result in slightly different GS. Moreover, the prostatectomy specimen is considered the diagnostic gold standard, which was used in only 55.6% of the investigated studies. Fourthly, no exact threshold values and sensitivity/specificity could be established for discrimination of clinical significant and non-significant cancers. This reflects one limitation of ADC values caused by variabilities due to hardware including different MRI scanners, sequence parameters, and interreader variability, which hinders to establish clear threshold values for clinical routine. However, as shown, the pooled ADC values of clinically insignificant PCs were no lower than 0.75 × 10^− 3^ mm^2^/s. Fifthly, our results might be affected by possible publication bias because negative studies, which could not identify an inverse correlation between PC with different GS might not be published.

Clearly, further prospective studies based on large samples are needed to proof and confirm our present results.

## Conclusion

Clinical significant PC showed lower ADC values compared to non-significant PC. The pooled ADC values of clinically insignificant PCs were no lower than 0.75 × 10^− 3^ mm^2^/s. This value may be proposed as a threshold for distinguishing clinically significant from insignificant PCs. The quantitative assessment of ADC should be included into the stratification of PCs in clinical practice.

## Data Availability

Data is available on reasonable request from the corresponding author.

## Abbreviations

mpMRI: multiparametric magnetic resonance imaging
PC: Prostate cancer
GS: Gleason score
DWI: Diffusion-weighted imaging
ADC: Apparent diffusion coefficient
PRISMA: Preferred Reporting Items for Systematic Reviews and Meta-Analyses
QUADAS: Quality Assessment of Diagnostic Accuracy Studies

## References

[1] Moldovan PC, Van den Broeck T, Sylvester R, Marconi L, Bellmunt J, van den RCN B, Bolla M, Briers E, Cumberbatch MG, Fossati N, Gross T, Henry AM, Joniau S, van der Kwast TH, Matveev VB, van der Poel HG, De Santis M, Schoots IG, Wiegel T, Yuan CY, Cornford P, Mottet N, Lam TB, Rouvière O (2017). What Is the Negative Predictive Value of Multiparametric Magnetic Resonance Imaging in Excluding Prostate Cancer at Biopsy? A Systematic Review and Meta-analysis from the European Association of Urology Prostate Cancer Guidelines Panel. Eur Urol.

[2] Ahmed HU, El-Shater Bosaily A, Brown LC, Gabe R, Kaplan R, Parmar MK, Collaco-Moraes Y, Ward K, Hindley RG, Freeman A, Kirkham AP, Oldroyd R, Parker C, Emberton M (2017). Diagnostic accuracy of multi-parametric MRI and TRUS biopsy in prostate cancer (PROMIS): a paired validating confirmatory study. Lancet..

[3] Greer MD, Brown AM, Shih JH, Summers RM, Marko J, Law YM, Sankineni S, George AK, Merino MJ, Pinto PA, Choyke PL, Turkbey B (2017). Accuracy and agreement of PIRADSv2 for prostate cancer mpMRI: a multireader study. J Magn Reson Imaging.

[4] Fütterer JJ, Briganti A, De Visschere P, Emberton M, Giannarini G, Kirkham A, Taneja SS, Thoeny H, Villeirs G, Villers A (2015). Can clinically significant prostate Cancer be detected with multiparametric magnetic resonance imaging? A systematic review of the literature. Eur Urol.

[5] Loeb S, Bjurlin MA, Nicholson J, Tammela TL, Penson DF, Carter HB, Carroll P, Etzioni R (2014). Overdiagnosis and overtreatment of prostate cancer. Eur Urol.

[6] Epstein JI, Egevad L, Amin MB, Delahunt B, Srigley JR, Humphrey PA (2016). The 2014 International Society of Urological Pathology (ISUP) consensus conference on Gleason grading of prostatic carcinoma: definition of grading patterns and proposal for a new grading system. Am J Surg Pathol.

[7] Nunez Bragayrac LA, Murekeyisoni C, Vacchio MJ, Attwood K, Mehedint DC, Mohler JL, Azabdaftari G, Xu B, Kauffman EC (2017). Blinded review of archival radical prostatectomy specimens supports that contemporary Gleason score 6 prostate cancer lacks metastatic potential. Prostate..

[8] Ham WS, Chalfin HJ, Feng Z, Trock BJ, Epstein JI, Cheung C, Humphreys E, Partin AW, Han M (2017). New prostate Cancer grading system predicts long-term survival following surgery for Gleason score 8-10 prostate Cancer. Eur Urol.

[9] Russo F, Regge D, Armando E, Giannini V, Vignati A, Mazzetti S, Manfredi M, Bollito E, Correale L, Porpiglia F (2016). Detection of prostate cancer index lesions with multiparametric magnetic resonance imaging (mp-MRI) using whole-mount histological sections as the reference standard. BJU Int.

[10] Dietrich O, Biffar A, Baur-Melnyk A, Reiser MF (2010). Technical aspects of MR diffusion imaging of the body. Eur J Radiol.

[11] Surov A, Meyer HJ, Wienke A (2017). Correlation between apparent diffusion coefficient (ADC) and cellularity is different in several tumors: a meta-analysis. Oncotarget..

[12] Hauge A, Wegner CS, Gaustad JV, Simonsen TG, Andersen LMK, Rofstad EK (2017). Diffusion-weighted MRI-derived ADC values reflect collagen I content in PDX models of uterine cervical cancer. Oncotarget..

[13] Koontz NA, Wiggins RH (2017). Differentiation of benign and malignant head and neck lesions with diffusion tensor imaging and DWI. AJR Am J Roentgenol.

[14] Suo S, Zhang K, Cao M, Suo X, Hua J, Geng X, Chen J, Zhuang Z, Ji X, Lu Q, Wang H, Xu J (2016). Characterization of breast masses as benign or malignant at 3.0T MRI with whole-lesion histogram analysis of the apparent diffusion coefficient. J Magn Reson Imaging.

[15] De Visschere PJ, Vral A, Perletti G, Pattyn E, Praet M, Magri V, Villeirs GM (2017). Multiparametric magnetic resonance imaging characteristics of normal, benign and malignant conditions in the prostate. Eur Radiol.

[16] Surov A, Meyer HJ, Wienke A (2019). Correlations between Apparent Diffusion Coefficient and Gleason Score in Prostate Cancer: A Systematic Review. Eur Urol Oncol.

[17] Moher D, Liberati A, Tetzlaff J, Altman DG (2009). Preferred reporting items for systematic reviews and meta-analyses: the PRISMA statement. PLoS Med.

[18] Bittencourt LK, Barentsz JO, de Miranda LC, Gasparetto EL (2012). Prostate MRI: diffusion-weighted imaging at 1.5T correlates better with prostatectomy Gleason grades than TRUS-guided biopsies in peripheral zone tumours. Eur Radiol.

[19] Caivano R, Rabasco P, Lotumolo A, Cirillo P, D'Antuono F, Zandolino A, Villonio A, Macarini L, Salvatore M, Cammarota A (2013). Comparison between Gleason score and apparent diffusion coefficient obtained from diffusion-weighted imaging of prostate cancer patients. Cancer Investig.

[20] Chatterjee A, Bourne RM, Wang S, Devaraj A, Gallan AJ, Antic T, Karczmar GS, Oto A (2018). Diagnosis of prostate Cancer with noninvasive estimation of prostate tissue composition by using hybrid multidimensional MR imaging: a feasibility study. Radiology..

[21] Chung MP, Margolis D, Mesko S, Wang J, Kupelian P, Kamrava M (2014). Correlation of quantitative diffusion-weighted and dynamic contrast-enhanced MRI parameters with prognostic factors in prostate cancer. J Med Imaging Radiat Oncol.

[22] Doo KW, Sung DJ, Park BJ, Kim MJ, Cho SB, Oh YW, Ko YH, Yang KS (2012). Detectability of low and intermediate or high risk prostate cancer with combined T2-weighted and diffusion-weighted MRI. Eur Radiol.

[23] Faletti R, Battisti G, Discalzi A, Grognardi ML, Martinello S, Oderda M, Gontero P, Bergamasco L, Cassinis MC, Fonio P (2016). Can DW-MRI, with its ADC values, be a reliable predictor of biopsy outcome in patients with suspected prostate cancer?. Abdom Radiol (NY).

[24] Glazer DI, Hassanzadeh E, Fedorov A, Olubiyi OI, Goldberger SS, Penzkofer T, Flood TA, Masry P, Mulkern RV, Hirsch MS, Tempany CM, Fennessy FM (2017). Diffusion-weighted endorectal MR imaging at 3T for prostate cancer: correlation with tumor cell density and percentage Gleason pattern on whole mount pathology. Abdom Radiol (NY).

[25] Hambrock T, Somford DM, Huisman HJ, van Oort IM, Witjes JA, Hulsbergen-van de Kaa CA, Scheenen T, Barentsz JO (2011). Relationship between apparent diffusion coefficients at 3.0-T MR imaging and Gleason grade in peripheral zone prostate cancer. Radiology..

[26] Ibrahiem EI, Mohsen T, Nabeeh AM, Osman Y, Hekal IA, Abou E-GM (2012). DWI-MRI: single, informative, and noninvasive technique for prostate cancer diagnosis. SciWorldJ..

[27] Li L, Margolis DJ, Deng M, Cai J, Yuan L, Feng Z, Min X, Hu Z, Hu D, Liu J, Wang L (2015). Correlation of Gleason scores with magnetic resonance diffusion tensor imaging in peripheral zone prostate cancer. J Magn Reson Imaging.

[28] Li C, Chen M, Wang J, Wang X, Zhang W, Zhang C (2017). Apparent diffusion coefficient values are superior to transrectal ultrasound-guided prostate biopsy for the assessment of prostate cancer aggressiveness. Acta Radiol.

[29] Liu W, Liu XH, Tang W, Gao HB, Zhou BN, Zhou LP (2018). Histogram analysis of stretched-exponential and monoexponential diffusion-weighted imaging models for distinguishing low and intermediate/high Gleason scores in prostate carcinoma. J Magn Reson Imaging.

[30] Nagarajan R, Margolis D, Raman S, Sheng K, King C, Reiter R, Thomas MA (2012). Correlation of Gleason scores with diffusion-weighted imaging findings of prostate cancer. Adv Urol.

[31] Park SY, Kim CK, Park JJ, Park BK (2016). Exponential apparent diffusion coefficient in evaluating prostate cancer at 3 T: preliminary experience. Br J Radiol.

[32] Sokmen BK, Sokmen D, Ucar N, Ozkurt H, Simsek A (2017). The correlation between biological activity and diffusion-weighted MR imaging and ADC value in cases with prostate cancer. Arch Ital Urol Androl.

[33] Somford DM, Hambrock T, Hulsbergen-van de Kaa CA, Fütterer JJ, van Oort IM, van Basten JP, Karthaus HF, Witjes JA, Barentsz JO (2012). Initial experience with identifying high-grade prostate cancer using diffusion-weighted MR imaging (DWI) in patients with a Gleason score ≤ 3 + 3 = 6 upon schematic TRUS-guided biopsy: a radical prostatectomy correlated series. Investig Radiol.

[34] Suo S, Chen X, Wu L, Zhang X, Yao Q, Fan Y, Wang H, Xu J (2014). Non-Gaussian water diffusion kurtosis imaging of prostate cancer. Magn Reson Imaging.

[35] Sprinkart AM, Marx C, Träber F, Block W, Thomas D, Schild H, Kukuk GM, Mürtz P (2018). Evaluation of exponential ADC (eADC) and computed DWI (cDWI) for the detection of prostate Cancer. Rofo..

[36] Tian W, Zhang J, Tian F, Shen J, Niu T, He G, Yu H (2018). Correlation of diffusion tensor imaging parameters and Gleason scores of prostate cancer. Exp Ther Med.

[37] Turkbey B, Shah VP, Pang Y, Bernardo M, Xu S, Kruecker J, Locklin J, Baccala AA, Rastinehad AR, Merino MJ, Shih JH, Wood BJ, Pinto PA, Choyke PL (2011). Is apparent diffusion coefficient associated with clinical risk scores for prostate cancers that are visible on 3-T MR images?. Radiology..

[38] Uribe CF, Jones EC, Chang SD, Goldenberg SL, Reinsberg SA, Kozlowski P (2015). In vivo 3T and ex vivo 7T diffusion tensor imaging of prostate cancer: correlation with histology. Magn Reson Imaging.

[39] Woo S, Kim SY, Cho JY, Kim SH (2016). Preoperative evaluation of prostate Cancer aggressiveness: using ADC and ADC ratio in determining Gleason score. AJR Am J Roentgenol.

[40] Wu X, Reinikainen P, Vanhanen A, Kapanen M, Vierikko T, Ryymin P, Hyödynmaa S, Kellokumpu-Lehtinen PL (2017). Correlation between apparent diffusion coefficient value on diffusion-weighted MR imaging and Gleason score in prostate cancer. Diagn Interv Imaging.

[41] Yağci AB, Ozari N, Aybek Z, Düzcan E (2011). The value of diffusion-weighted MRI for prostate cancer detection and localization. Diagn Interv Radiol.

[42] Zhang YD, Wang Q, Wu CJ, Wang XN, Zhang J, Liu H, Liu XS, Shi HB (2015). The histogram analysis of diffusion-weighted intravoxel incoherent motion (IVIM) imaging for differentiating the Gleason grade of prostate cancer. Eur Radiol.

[43] Zhang Z, Xu H, Xue Y, Li J, Ye Q (2019). Risk stratification of prostate Cancer using the combination of histogram analysis of apparent diffusion coefficient across tumor diffusion volume and clinical information: a pilot study. J Magn Reson Imaging.

[44] Whiting PF, Rutjes AW, Westwood ME, Mallett S, Deeks JJ, Reitsma JB (2011). QUADAS-2: a revised tool for the quality assessment of diagnostic accuracy studies. Ann Intern Med.

[45] Leeflang MM, Deeks JJ, Gatsonis C, Bossuyt PM (2008). Systematic reviews of diagnostic test accuracy. Ann Intern Med.

[46] Zamora J, Abraira V, Muriel A, Khan K, Coomarasamy A (2006). Meta-DiSc: a software for meta-analysis of test accuracy data. BMC Med Res Methodol.

[47] DerSimonian R, Laird N (1986). Meta-analysis in clinical trials. Control Clin Trials.

[48] Pierorazio PM, Walsh PC, Partin AW, Epstein JI (2013). Prognostic Gleason grade grouping: data based on the modified Gleason scoring system. BJU Int.

[49] Miyake H, Muramaki M, Furukawa J, Tanaka H, Inoue TA, Fujisawa M (2013). Prognostic significance of primary Gleason pattern in Japanese men with Gleason score 7 prostate cancer treated with radical prostatectomy. Urol Oncol.

[50] Bourne R, Panagiotaki E (2016). Limitations and Prospects for Diffusion-Weighted MRI of the Prostate. Diagnostics (Basel).

[51] Jambor I (2017). Optimization of prostate MRI acquisition and post-processing protocol: a pictorial review with access to acquisition protocols. Acta Radiol Open.

[52] Merisaari H, Movahedi P, Perez IM, Toivonen J, Pesola M, Taimen P, Boström PJ, Pahikkala T, Kiviniemi A, Aronen HJ, Jambor I (2017). Fitting methods for intravoxel incoherent motion imaging of prostate cancer on region of interest level: repeatability and Gleason score prediction. Magn Reson Med.

[53] Chatterjee A, Watson G, Myint E, Sved P, McEntee M, Bourne R (2015). Changes in epithelium, Stroma, and lumen space correlate more strongly with Gleason pattern and are stronger predictors of prostate ADC changes than cellularity metrics. Radiology..

[54] Ma T, Yang S, Jing H, Cong L, Cao Z, Liu Z, Huang Z (2018). Apparent diffusion coefficients in prostate cancer: correlation with molecular markers Ki-67, HIF-1α and VEGF. NMR Biomed.

[55] Shaish H, Kang SK, Rosenkrantz AB (2017). The utility of quantitative ADC values for differentiating high-risk from low-risk prostate cancer: a systematic review and meta-analysis. Abdom Radiol (NY)..

[56] Jambor I, Merisaari H, Taimen P, Boström P, Minn H, Pesola M, Aronen HJ (2015). Evaluation of different mathematical models for diffusion-weighted imaging of normal prostate and prostate cancer using high b-values: a repeatability study. Magn Reson Med.

[57] Merisaari H, Toivonen J, Pesola M, Taimen P, Boström PJ, Pahikkala T, Aronen HJ, Jambor I (2015). Diffusion-weighted imaging of prostate cancer: effect of b-value distribution on repeatability and cancer characterization. Magn Reson Imaging.

[58] Huang CC, Kong MX, Zhou M, Rosenkrantz AB, Taneja SS, Melamed J, Deng FM (2014). Gleason score 3 + 4=7 prostate cancer with minimal quantity of Gleason pattern 4 on needle biopsy is associated with low-risk tumor in radical prostatectomy specimen. Am J Surg Pathol.

[59] Stark JR, Perner S, Stampfer MJ, Sinnott JA, Finn S, Eisenstein AS, Ma J, Fiorentino M, Kurth T, Loda M, Giovannucci EL, Rubin MA, Mucci LA (2009). Gleason score and lethal prostate cancer: does 3+4=4+3?J. Clin Oncol.

[60] Makarov DV, Sanderson H, Partin AW, Epstein JI (2002). Gleason score 7 prostate cancer on needle biopsy: is the prognostic difference in Gleason scores 4+3 and 3+4 independent of the number of involved cores?. J Urol.

[61] Amin A, Partin A, Epstein JI (2011). Gleason score 7 prostate cancer on needle biopsy: relation of primary pattern 3 or 4 to pathological stage and progression after radical prostatectomy. J Urol.

[62] Matoso A, Epstein JI (2019). Defining clinically significant prostate cancer on the basis of pathological findings. Histopathology.

[63] Slaoui H, Neuzillet Y, Ghoneim T, Rouanne M, Abdou A, Lugagne-Delpon PM, Scherrer A, Radulescu C, Delancourt C, Molinié V, Lebret T (2017). Gleason score within prostate abnormal areas defined by multiparametric magnetic resonance imaging did not vary according to the PIRADS score. Urol Int.

[64] Pierre T, Cornud F, Colléter L, Beuvon F, Foissac F, Delongchamps NB, Legmann P (2018). Diffusion-weighted imaging of the prostate: should we use quantitative metrics to better characterize focal lesions originating in the peripheral zone?. Eur Radiol.

